# The Activities of the Gelsolin Homology Domains of Flightless-I in Actin Dynamics

**DOI:** 10.3389/fmolb.2020.575077

**Published:** 2020-09-08

**Authors:** Réka Pintér, Tamás Huber, Péter Bukovics, Péter Gaszler, Andrea Teréz Vig, Mónika Ágnes Tóth, Gabriella Gazsó-Gerhát, Dávid Farkas, Ede Migh, József Mihály, Beáta Bugyi

**Affiliations:** ^1^Department of Biophysics, Medical School, University of Pécs, Pécs, Hungary; ^2^Biological Research Centre Szeged, Institute of Genetics, Szeged, Hungary; ^3^Faculty of Science and Informatics, Doctoral School in Biology, University of Szeged, Szeged, Hungary; ^4^Szentágothai Research Center, Pécs, Hungary

**Keywords:** actin, gelsolin homology, Flightless-I, *Drosophila*, fluorescence

## Abstract

Flightless-I is a unique member of the gelsolin superfamily alloying six gelsolin homology domains and leucine-rich repeats. Flightless-I is an established regulator of the actin cytoskeleton, however, its biochemical activities in actin dynamics are still largely elusive. To better understand the biological functioning of Flightless-I we studied the actin activities of *Drosophila* Flightless-I by *in vitro* bulk fluorescence spectroscopy and single filament fluorescence microscopy, as well as *in vivo* genetic approaches. Flightless-I was found to interact with actin and affects actin dynamics in a calcium-independent fashion *in vitro*. Our work identifies the first three gelsolin homology domains (1–3) of Flightless-I as the main actin-binding site; neither the other three gelsolin homology domains (4–6) nor the leucine-rich repeats bind actin. Flightless-I inhibits polymerization by high-affinity (∼nM) filament barbed end capping, moderately facilitates nucleation by low-affinity (∼μM) monomer binding, and does not sever actin filaments. Our work reveals that in the presence of profilin Flightless-I is only able to cap actin filament barbed ends but fails to promote actin assembly. In line with the *in vitro* data, while gelsolin homology domains 4–6 have no effect on *in vivo* actin polymerization, overexpression of gelsolin homology domains 1–3 prevents the formation of various types of actin cables in the developing *Drosophila* egg chambers. We also show that the gelsolin homology domains 4–6 of Flightless-I interact with the C-terminus of *Drosophila* Disheveled-associated activator of morphogenesis formin and negatively regulates its actin assembly activity.

## Introduction

**FIGURE 1:**
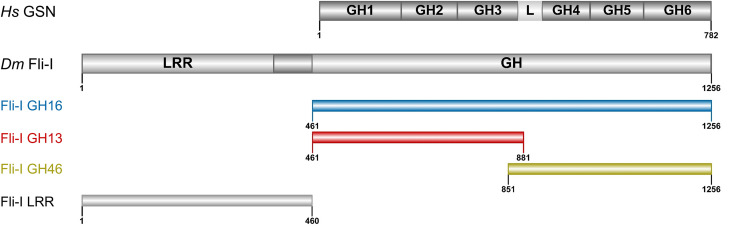
Domain organization of gelsolin and Flightless-I. Domain organization of gelsolin (GSN: 1–782 aa) and the Flightless-I constructs used in our study (Fli-I GH16: 461–1256 aa, GH13: 461–881 aa, GH46: 851–1256 aa, LRR: 1–460 aa). The Figure was made by IBS 1.0.2 (Liu et al., 2015). *Hs, Homo sapiens; Dm, Drosophila melanogaster*; GSN, gelsolin; Fli-I, Flightless-I; GH, gelsolin homology; L, GH3-GH4 linker region; LRR, leucine-rich repeat.

The gelsolin (GSN) superfamily comprises actin-remodeling proteins including gelsolin, Flightless-I (Fli-I), villin, adseverin, macrophage capping protein (CapG), advillin and supervillin that regulate diverse aspects of the actin cytoskeleton (reviewed in Burtnick et al., 2001; Silacci et al., 2004; Ghoshdastider et al., 2013; Nag et al., 2013). The eponymous member; gelsolin with six gelsolin domains (Figure 1) is a Ca^2+^-regulated multifunctional protein; it interacts with both filamentous and monomeric actin and possesses barbed end capping, severing and nucleation activities *in vitro* (Burtnick et al., 2001; Silacci et al., 2004; Nag et al., 2013).

Fli-I was originally characterized in *Drosophila melanogaster* as a protein product of the *flightless-I* gene associated with developmental processes including cellularization and organization of indirect flight muscle (Perrimon et al., 1989; Campbell et al., 1993). Disruption of *fli-I* can cause lethality in early embryogenesis and defects in actin-associated processes in mouse, *Drosophila* and *C. elegans* indicating the essential role of the protein in embryonic development (Campbell et al., 1993; Campbell et al., 2002; Deng et al., 2007; Lu et al., 2008). In contrast, homozygous null gelsolin (Witke et al., 1995), CapG (Witke et al., 2001) or villin (Pinson et al., 1998; Ferrary et al., 1999) mutant mice are viable and fertile. The vital role of Fli-I is further supported by its tissue distribution that is the most widespread amongst GSN family proteins; it is abundantly expressed in skeletal and heart muscles, as well as in nerve cells (Campbell et al., 1993; Campbell et al., 1997; Davy et al., 2000; Nag et al., 2013). Fli-I is a well-established negative regulator of wound healing and tissue regeneration (Cowin et al., 2007; Cameron et al., 2016). The human protein is implicated in epidermolysis bullosa and Smith-Magenis syndrome causing developmental and behavioral abnormalities (Chen et al., 1995; Kopecki et al., 2011).

Fli-I alloys domains from two protein families that endows it with unique structural characteristics (Figure 1). The N-terminal region is composed of tandem leucine-rich repeats (LRRs) forming a protein-protein interaction domain. The C-terminal half of the protein possesses six gelsolin homology (GH) domains analogously to gelsolin. The LRR region of Fli-I has diverse interaction partners and may participate in interconnecting signaling and cytoskeletal reorganization events (Liu and Yin, 1998; Fong and de Couet, 1999; Goshima et al., 1999; Davy et al., 2000), while the C-terminal GH domains are thought to serve as a platform for actin interactions. Notably, the actin-binding ability of Fli-I of human, mouse and *C. elegans* origin has been demonstrated *in vitro* by pull-down and sedimentation approaches in cell extracts, as well as with purified proteins (Liu and Yin, 1998; Goshima et al., 1999; Li et al., 2008; Mohammad et al., 2012). The association of Fli-I to actin-based structures was confirmed in various cell lines and also in animal models (mouse, *Drosophila, C. elegans*) (Davy et al., 2000, 2001; Campbell et al., 2002; Deng et al., 2007; Li et al., 2008; Lu et al., 2008; Mohammad et al., 2012). The binding of Fli-I to both G-actin and F-actin was suggested (Liu and Yin, 1998; Goshima et al., 1999; Mohammad et al., 2012). The respective contribution of the GH domains of Fli-I to actin-binding has not been investigated, also its binding strengths to G-, and F-actin are not known.

The actin interactions and activities of Fli-I do not seem to rely on calcium *in vitro*, suggesting its different mode of regulation comparing against the calcium-dependent activation of gelsolin (Goshima et al., 1999; Mohammad et al., 2012). Biochemical analysis revealed that Fli-I retards actin assembly in bulk pyrenyl polymerization experiments and increases the amount of unassembled actin at steady-state, leading to the suggestion that Fli-I acts as a capping protein (Mohammad et al., 2012; Arora et al., 2015). The filament severing activity of Fli-I was proposed based on the appearance of short actin filaments in electron microscopy images in the presence of Fli-I (Goshima et al., 1999). Actively promoting F-actin disassembly by severing is expected to accelerate filament disassembly kinetics in dilution induced bulk depolymerization experiments (Coue and Korn, 1985; Kinosian et al., 1998; Toth et al., 2016). In contrast, Fli-I failed to enhance the rate of filament disassembly in such assays (Mohammad et al., 2012). Thus, albeit several studies have already been performed regarding the effects of Fli-I on actin dynamics, conflicting data exist in the literature; the actin activities of Fli-I and the underlying mechanisms are still largely elusive. To get further insights into the biological functioning of Fli-I we aimed to analyze the biochemical activities of recombinantly produced *Drosophila* proteins including the gelsolin homology domains, as well as the leucine-rich repeat segment. We took advantage of the combination of bulk fluorescence spectroscopy and individual filament total internal reflection fluorescence microscopy (TIRFM) approaches to dissect the activities of different regions of Fli-I in the regulation of actin dynamics. We also investigated the influence of Fli-I on actin cytoskeleton in developing *Drosophila* egg chambers by *in vivo* genetic approaches.

## Results

### The Gelsolin Homology Domains of Fli-I Interact With Actin and Affect Actin Dynamics in a Calcium-Independent Manner

**FIGURE 2:**
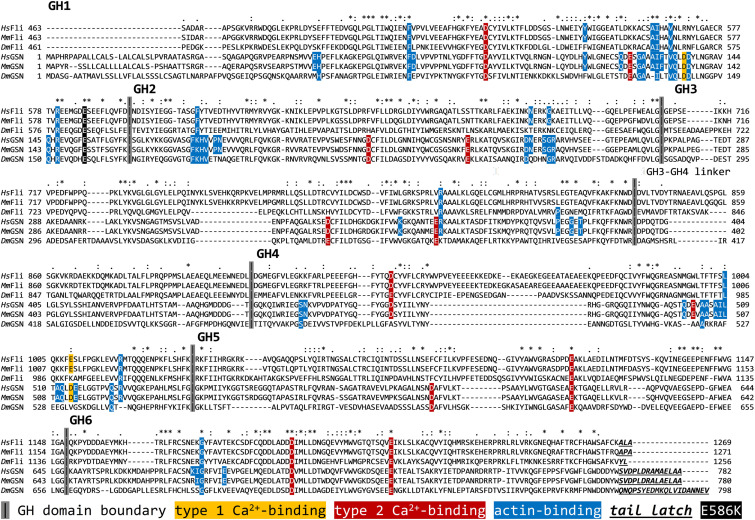
Comparative sequence analysis of the gelsolin homology domains of Fli-I and gelsolin from different species. UniProt IDs: Q13045 (*Hs* Fli-I), Q9JJ28 (*Mm* Fli-I), Q24020 (*Dm* Fli-I), P06396 (*Hs* GSN), P13020 (*Mm* GSN), Q07171 (*Dm* GSN). The analysis was performed by ClustalX. *Hs, Homo sapiens; Mm, Mus musculus; Dm, Drosophila melanogaster*; FliI, Flightless-I; GSN, gelsolin; GH, gelsolin homology.

**FIGURE 3:**
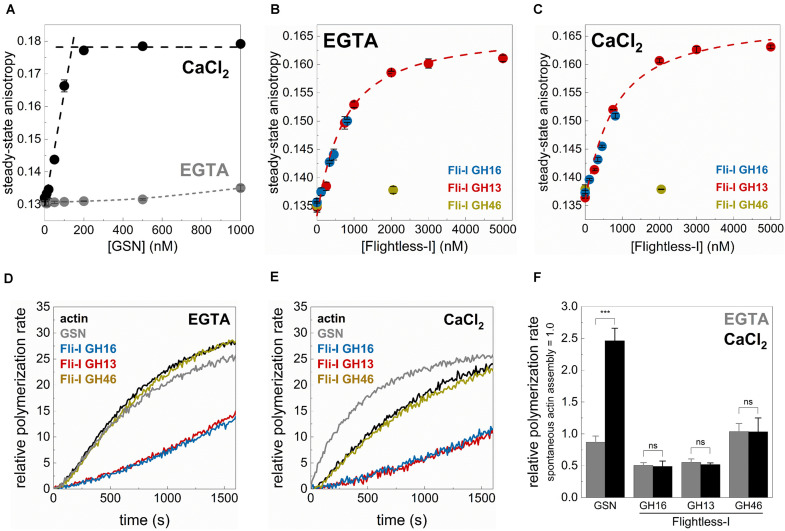
The gelsolin homology domains of Flightless-I influence actin dynamics in a Ca^2+^-independent manner. **(A)** Steady-state anisotropy of Alexa488NHS-G-actin (0.2 μM) as a function of [GSN] in the presence of 1 mM EGTA (Ca^2+^-free condition) or 1 mM CaCl_2_. Black dashed lines show the linear fits to the data measured at low and high [GSN]. The breakpoint was found at [GSN] = 140.05 nM indicating a ∼1 GSN:2 G-actin stoichiometry. Data are shown as mean ± SD, *n* = 3. **(B,C)** Steady-state anisotropy of Alexa488NHS-G-actin (0.2 μM) as a function of [GST-Fli-I] in the presence of 1 mM EGTA (Ca^2+^-free condition) **(B)** or 1 mM CaCl_2_ **(C)**. Red dashed lines show the fit of the data according to Eq. 3. The fit gave the following dissociation equilibrium constants: K_D(Fli–I GST–GH13)_ = 576 ± 182 nM (1 mM EGTA), K_D(Fli–I GST–GH13)_ = 638 ± 152 nM (1 mM CaCl_2_). Data are shown as mean ± SD, *n* = 3. **(D,E)** Representative pyrenyl emission kinetics recorded in the absence or presence of GST-Fli-I (5 nM) or GSN (5 nM) and in the presence of 1 mM EGTA **(D)** or 1 mM CaCl_2_ **(E)**. Conditions: 2.5 μM actin (5% pyrenyl labeled). **(F)** Relative polymerization rates derived from pyrenyl transients shown on panels **(D,E)**. Data are shown as mean ± SD, *n* = 2–4. ***p < 0.001.

Calcium-binding of full-length gelsolin is a prerequisite for the activation of its actin interactions and activities (Burtnick et al., 2001; Silacci et al., 2004; Nag et al., 2013; Feldt et al., 2019). Proteolytic cleavage of the protein at the caspase 3 site results in a Ca^2+^-independent N-terminal (GH13) and a Ca^2+^ dependent C-terminal (GH46) halves (Figure 1) (Pope et al., 1991; Kothakota et al., 1997; Silacci et al., 2004; Nag et al., 2013). Comparative sequence analysis of gelsolin and Fli-I reveals that most of the sequence elements responsible for the Ca^2+^-induced activation of gelsolin (C-terminal helical latch, type 1 and type 2 Ca^2+^-binding sites) are not conserved in Fli-I (Figure 2; Goshima et al., 1999; Nag et al., 2013). Previous work did not find any effect of Ca^2+^ on the actin interactions of Fli-I proteins from mouse and *C. elegans*, supporting that the actin activities of Fli-I do not rely on Ca^2+^-binding (Goshima et al., 1999; Mohammad et al., 2012). Based on the above, first we investigated the actin interactions and activities of *Drosophila* Fli-I in the absence and presence of calcium ions (Figure 3). A fragment of Fli-I encompassing all the six gelsolin homology domains (GST-GH16) was produced recombinantly as a GST fusion protein, similar to previous approaches (Goshima et al., 1999; Li et al., 2008; Mohammad et al., 2012; Figure 1). To dissect the activities of the different regions of the protein, an N- (GST-GH13), and a C-terminal (GST-GH46) GST-tagged segment corresponding to the caspase 3 proteolytic fragments of gelsolin, as well as the isolated leucine-rich repeat region (GST-LRR) were investigated (Figure 1).

Gelsolin is known to bind monomeric actin and forms a GA_2_ (1 GSN:2 G-actin) complex in a Ca^2+^-dependent fashion (Coue and Korn, 1985; Selden et al., 1998; Nag et al., 2013). Consistently, by monitoring the steady-state anisotropy of fluorescently labeled G-actin (0.2 μM Alexa488NHS-G-actin) we found that GSN binds weakly to monomeric actin in the absence of Ca^2+^ (1 mM EGTA condition), while the addition of CaCl_2_ (1 mM) profoundly strengthens the interaction (Figure 3A). The break-point titration is consistent with the 1 GSN:2 G-actin stoichiometry, as well as with the high-affinity of the complex. The addition of Fli-I GST-GH16 to monomeric actin, even in the absence of CaCl_2_ (1 mM EGTA condition) resulted in a significant increase in anisotropy from ∼0.136 (in the absence of GST-GH16) to ∼0.150 (in the presence of ∼1 μM GST-GH16; the maximum amount of protein that could be tested in these experiments) (Figure 3B). This result suggests a direct binding between the gelsolin homology domains of Fli-I and G-actin in agreement with previous reports (Liu and Yin, 1998; Goshima et al., 1999; Mohammad et al., 2012). A similar response in a broader concentration range could be detected in the case of Fli-I GST-GH13 (Figure 3B). The fit of the anisotropy data gave a dissociation equilibrium constant of K_D(Fli–I GST–GH13)_ = 576 ± 182 nM of the Fli-I GST-GH13:G-actin complex (Figure 3B, Eq. 3). Analysis of the [GST-Fli-I] dependence of the anisotropy measured in the presence of CaCl_2_ (1 mM) revealed a similar binding trend and affinity as detected in the absence of the divalent cation, indicating that the interaction of Fli-I with G-actin is not affected by the presence of Ca^2+^ (K_D(Fli–I GST–GH13)_ = 638 ± 152 nM, Figure 3C). No significant change in anisotropy was found when Fli-I GST-GH46 (∼2 μM) was added to G-actin; either in EGTA or CaCl_2_ conditions suggesting the lack of actin interaction of this region (Figures 3B,C).

Subsequently, the calcium-response of the effects of Fli-I on actin assembly was monitored in pyrenyl polymerization experiments (Figures 3D–F). In control measurements, we found that gelsolin (5 nM) does not significantly affect actin polymerization in a Ca^2+^-free environment (1 mM EGTA condition), while it accelerates actin assembly kinetics in the presence of CaCl_2_ (1 mM) (2.46 ± 0.19-fold increase, *n* = 2–3, *p* = 0.001) (Figures 3D–F; Yin and Stossel, 1979; Coue and Korn, 1985; Kis-Bicskei et al., 2018). In contrast to the calcium-dependent polymerization promoting effect of GSN, Fli-I GST-GH16 (5 nM) inhibited actin assembly to the same extent both in the absence and presence of Ca^2+^ (*n* = 2–3, *p* = 0.554) (Figures 3D–F). The inhibitory effect of Fli-I on the rate of actin polymer formation is in agreement with previous reports (Mohammad et al., 2012). Qualitatively and quantitatively the same response was detected for Fli-I GST-GH13 (5 nM) (Figures 3D–F). Whereas Fli-I GST-GH46 (5 nM) did not have any effect on actin assembly, independently from the presence of calcium (*n* = 3, *p* = 0.976) (Figures 3D–F).

Altogether, these observations support that in contrast to gelsolin, the actin-related activities of Fli-I are not regulated by Ca^2+^-binding. On the other hand, it is important to note that the GH13 region of Fli-I seems to be responsible for the G-actin interaction and the actin assembly inhibition activities of the gelsolin homology domains of the protein.

### The Gelsolin Homology Domains of Fli-I Affect Actin Assembly From Free G-Actin That Relies on the GH13 Regions

**FIGURE 4:**
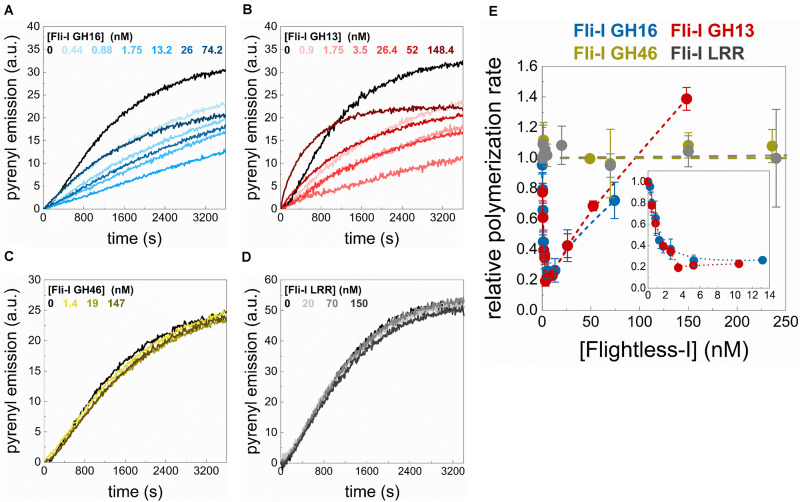
The gelsolin homology domains of Flightless-I affect actin assembly that relies on its GH13 domains. **(A–D)** Representative pyrenyl fluorescence emission kinetics recorded in the absence or presence of different concentrations of GST-Fli-I constructs. Conditions: 2.5 μM actin (5% pyrenyl labeled). **(E)** Relative polymerization rate as a function of [GST-Fli-I]. Data are shown as mean ± SD, *n* = 2–7. Inset: enlarged view of the data corresponding to low [GST-Fli-I] (<14 nM).

To address the biochemical activities of Fli-I in actin dynamics, the effects of Fli-I on actin assembly kinetics from free G-actin were further investigated in bulk pyrenyl polymerization experiments (Figure 4). The data revealed that the effects of Fli-I GST-GH16 on polymer formation from free G-actin follow a biphasic concentration-response. At lower concentrations (<∼10 nM) it inhibits actin polymerization; in contrast, at higher concentrations of Fli-I GST-GH16 (>∼25 nM) the inhibition was less pronounced (Figures 4A,E). We found that Fli-I GST-GH13 can influence actin dynamics in a qualitatively and quantitatively similar manner as Fli-I GST-GH16 (Figures 4B,E). Larger concentrations of GST-GH13 (>∼100 nM) even accelerates polymerization above the spontaneous rate (Figures 4B,E). Based on the tendency; this behavior would be expected of GST-GH16 if higher concentrations could be tested in the experiments. In contrast, the GST-GH46 region does not affect actin polymerization in the concentration range in which the two other fragments of Fli-I were tested (Figures 4C,E). These results are consistent with our previous data and demonstrate that the actin activities of Fli-I GST-GH16 are reconstituted by the GST-GH13 segment, while the GST-GH46 region is not able to interact with actin. Moreover, the biphasic nature of the effect of Fli-I GST-GH13/GST-GH16 on polymerization kinetics suggests multiple activities in actin dynamics.

We also tested whether the isolated leucine-rich repeat of Fli-I interacts with actin. In steady-state anisotropy measurements, no significant change was detected upon titration of G-actin (0.2 μM Alexa488NHS-G-actin) with Fli-I GST-LRR (*r* = 0.130 ± 0.001 in the absence of GST-LRR and *r* = 0.130 ± 0.001 in the presence of 800 nM GST-LRR). Also, Fli-I GST-LRR does not affect actin assembly in bulk pyrenyl polymerization experiments (Figures 4D,E). These observations indicate that similarly to GH46, the isolated LRR does not interact with actin (Liu and Yin, 1998).

### The Gelsolin Homology Domains of Fli-I Inhibit Actin Filament Growth by Barbed End Capping

The polymerization inhibition that we observe at low nM concentrations of Fli-I reflects high-affinity interactions and can result from the prevention of subunit addition to filament ends. This can be manifested by capping through filament end interactions, but also by sequestration upon binding to monomeric actin. On the other hand, some proteins by interacting with fluorescently labeled actin (e.g., by pyrene, IAEDANS) can modify its structural properties resulting in a change in the spectral characteristics of the actin-bound fluorophore (e.g., Leiomodin (Lmod), Wiskott-Aldrich Syndrome Homology 2 domains of Sarcomere Length Short (SALS-WH2) (Toth et al., 2016; Szatmari et al., 2017). This effect could result in an apparent change in pyrenyl kinetics even in the absence of any functional effects on actin polymerization.

**FIGURE 5:**
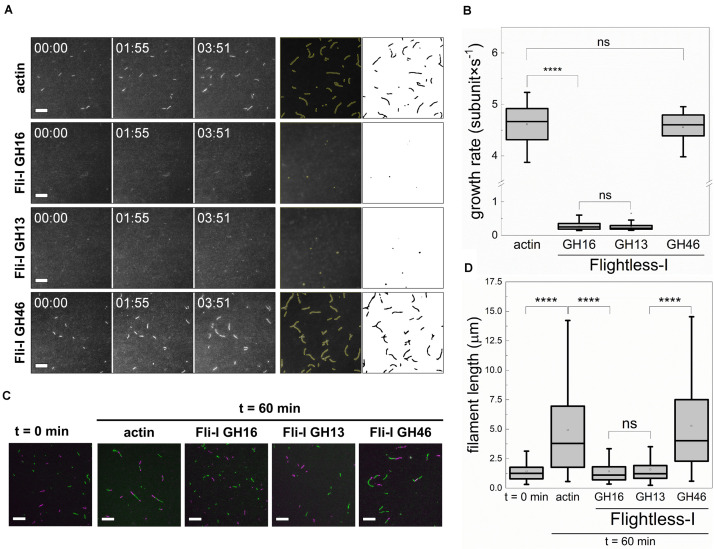
The gelsolin homology domains of Flightless-I inhibit actin filament growth by barbed end capping. **(A)** Left panels: Representative montages of actin assembly followed by TIRFM in the absence or presence of GST-Fli-I. Right panels: Representative skeletonized TIRFM images used for analysis showing the field of view of a 66 × 66 μm^2^ region in the absence or presence of GST-Fli-I. Conditions: [actin] = 0.5 μM (10% Alexa488NHS labeled), [Fli-I GST-GH16] = 10 nM, [Fli-I GST-GH13] = 10 nM, [Fli-I GST-GH46] = 100 nM. Scale bar = 10 μm, time = min:s. **(B)** Filament growth rate from G-actin in the absence or presence of different GST-Fli-I constructs derived from time-lapse TIRFM images shown on **(A)**, *n* = 20–60. **(C)** Representative montage of actin filament end-to-end annealing followed by TIRFM in the absence or presence of different GST-Fli-I constructs. The *t* = 0 min corresponds to the initial fragmentation of filaments, while *t* = 60 min indicates the time interval after fragmentation. Conditions: [actin] = 0.5 μM (10% Alexa488NHS and 10% Alexa568NHS labeled), [GST-Fli-I] = 120 nM. Scale bar = 10 μm. **(D)** Filament length at *t* = 0 min and 60 min in the absence or presence of GST-Fli-I, derived from TIRFM images shown on **(C)**, n_*samples*_ = 3–9, n_Filaments_ = 136–174. ****p < 0.0001.

To elaborate on the mechanisms underlying the polymerization inhibition activity of Fli-I, actin assembly was visualized at the level of individual polymers by using total internal reflection fluorescence microscopy (Figures 5A,B). In control samples, polymers were nucleated spontaneously and elongated at a rate of v = 4.61 ± 0.36 subunit × s^–1^ (*n* = 23) that corresponds to the well-established barbed end association rate constant of free G-actin (k_+_ = 11.53 ± 0.90 μM^–1^s^–1^; Pollard, 2007; Bugyi and Carlier, 2010). Addition of Fli-I GST-GH16 (10 nM) to actin resulted in almost complete inhibition of polymer growth (0.24 ± 0.10 subunit × s^–1^, *n* = 39, *p* ≤ 0.0001). Consistently with the observations made in the fluorescence spectroscopy experiments, the effects of Fli-I GST-GH16 on polymer assembly can be recapitulated by Fli-I GST-GH13; as 10 nM GST-GH13 resulted in a similar inhibition as observed for 10 nM GST-GH16 (v = 0.28 ± 0.12 subunit × s^–1^, *n* = 33, *p* = 0.158). Also, we found that Fli-I GST-GH46 does not have significant effect on polymer growth rate in TIRFM assays (v = 4.56 ± 0.30 subunit × s^–1^, *n* = 20, *p* = 0.589). Altogether, the TIRFM data support the results obtained from pyrenyl fluorescence experiments: low amounts of Fli-I inhibits actin assembly and this activity relies on the GH13 segment of the protein.

Based on the experimental conditions in our TIRFM assays (0.5 μM free G-actin; below the pointed end critical concentration), actin polymer growth is dominated by barbed end assembly. Considering the dissociation equilibrium constant of the GST-Fli-I:G-actin interaction derived from anisotropy measurements (Figures 3B,C), 10 nM Fli-I – which causes polymer growth inhibition in TIRFM experiments – is expected to bind to ∼1% of the G-actin; i.e., ∼5 nM. This would result in a negligible reduction (∼1.4%) in the polymer growth rate as predicted by Eq. 4. Thus, G-actin sequestration that relies on monomer binding by Fli-I does not explain the marked polymerization inhibition observed at nanomolar protein concentrations. Consequently, our data point toward barbed end related activities. To experimentally address this issue, the end-to-end annealing of preformed, mechanically fragmented filaments was monitored by dual-color TIRFM (Figures 5C,D). In the absence of Fli-I the spontaneous lengthwise association, i.e., annealing of the actin filament fragments was supported by the increase in filament length with time, as well as by the appearance of spectrally inhomogeneous filaments. In the presence of either Fli-I GST-GH16 or GST-GH13 (120 nM) the filaments remained short and were characterized by homogeneous fluorescence emission indicating that annealing is inhibited by these constructs. No such inhibitory effect was detected when Fli-I GST-GH46 was added to actin, in agreement with our previous observations.

Collectively, these data show that Fli-I possesses a filament end capping activity and support that the inhibition of actin assembly by Fli-I results from the prevention of actin incorporation at the barbed ends.

### The Gelsolin Homology Domains of Fli-I Do Not Sever Filaments but Facilitate the Formation of Nucleation Intermediates

**FIGURE 6:**
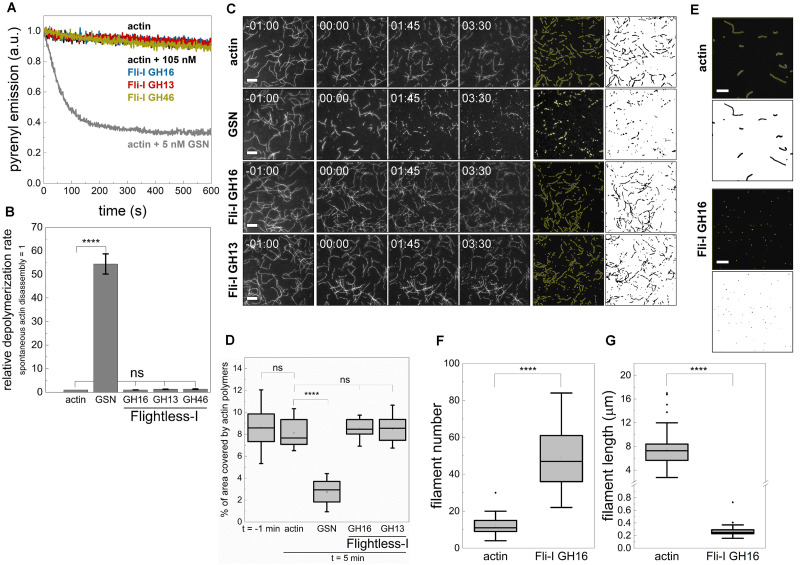
The gelsolin homology domains of Flightless-I do not sever filaments but facilitate the formation of nucleation intermediates. **(A)** Kinetics of actin polymer disassembly as followed by the decrease in pyrenyl fluorescence emission in the absence or presence of GST-Fli-I or GSN. Conditions: [actin] = 50 nM (50% pyrenyl labeled), [GSN] = 5 nM, [GST-Fli-I] = 105 nM, [CaCl_2_] = 1 mM. Note that the depolymerization of F-actin in the absence (actin; black line) and presence of different GST-Fli-I constructs (105 nM; blue, red and yellow traces, as indicated) follow similar kinetics; the pyrenyl traces largely overlap. While that of recorded in the presence of GSN (5 nM; gray) differs significantly. **(B)** Relative depolymerization rate derived from the pyrenyl transients shown on panel **(A)**, *n* = 2–3. **(C)** Left panels: Representative montages of actin disassembly followed by TIRFM in the absence or presence of GST-Fli-I or GSN. The frames labeled by -01:00 and 00:00 show the field of view just before and right after the addition of GSN or GST-Fli-I, respectively. Right panels: Representative skeletonized TIRFM images used for filament area analysis showing the field of view of a 66 × 66 μm^2^ region in the absence or presence of GSN or GST-Fli-I. Conditions: [actin] = 0.5 μM (10% Alexa488NHS labeled), [GSN] = 0.5 nM, [GST-Fli-I] = 105 nM. Scale bar = 10 μm, time = min:s. **(D)** Percent of the area covered by actin filaments 5 min after the addition of GSN or GST-Fli-I derived from TIRFM images shown on **(C)**, *n* = 12–51. **(E)** Representative skeletonized TIRFM images used for filament number and length analysis showing the field of view of a 66 × 66 μm^2^ region in the absence or presence of Fli-I GST-GH16. Conditions: [actin] = 2 μM (10% Alexa488NHS labeled), [Fli-I GST-GH16] = 800 nM. Scale bar = 10 μm. **(F)** Number of filaments assembled from G-actin either spontaneously or in the presence of Fli-I GST-GH16 derived from TIRFM images shown on **(E)**, *n* = 69–73. **(G)** Filament length assembled from G-actin either spontaneously or in the presence of Fli-I GST-GH16 derived from TIRFM images shown on **(E)**, *n* = 69–73. ****p < 0.0001.

Pyrenyl polymerization experiments revealed that the addition of larger amounts of Fli-I resulted in facilitated polymerization (Figures 4A,B,E) that might result from cutting of the actin filaments thereby generating more ends for elongation (severing) and/or enhanced nucleation. To test the severing ability of Fli-I, dilution-induced bulk disassembly kinetics measurements were performed (Figures 6A,B). In control experiments we found that the spontaneous disassembly of actin filaments is relatively slow, in contrast, gelsolin (5 nM, in the presence of 1 mM CaCl_2_) accelerated disassembly kinetics by ∼50-fold consistently with its severing activity (Figures 6A,B; Kinosian et al., 1998; Nag et al., 2013; Toth et al., 2016). In the presence of GST-Fli-I at a concentration that can enhance actin polymerization in pyrenyl fluorescence experiments (105 nM, Figure 4E) no significant increase in the rate of filament disassembly was observed as compared to spontaneous depolymerization, neither in the absence (*data not shown*) nor in the presence of 1 mM CaCl_2_ (Figures 6A,B). As an alternative approach, the disassembly efficiency of gelsolin and GST-Fli-I was also visualized in TIRFM experiments by adding the proteins to preassembled filaments (Figures 6C,D). The disassembly activity was quantified by measuring the area covered by filamentous actin after 5 min following gelsolin or GST-Fli-I addition. The presence of gelsolin (0.5 nM, in the presence of 1 mM CaCl_2_) resulted in a marked decrease in the filament area as compared to the control (A_actin_ = 337.47 ± 84.92 μm^2^, n = 10, A_GSN_ = 165.95 ± 49.05 μm^2^, *n* = 12, *p* ≤ 0.0001). In contrast, addition of either the GST-GH16 or GST-GH13 fragments of Fli-I (105 nM) did not influence significantly this parameter (A_Fli–I GST–GH16_ = 373.44 ± 38.458 μm^2^, *n* = 14, *p* = 0.224, A_Fli–I GST–GH13_ = 370.31 ± 54.58 μm^2^, *n* = 12, *p* = 0.373). Based on these results, we conclude that, in contrast to gelsolin, the gelsolin homology domains of Fli-I do not possess actin filament severing activity.

Considering the monomer binding ability of Fli-I revealed by anisotropy measurements, we hypothesized that the assembly promoting activity of Fli-I results from its ability to *de novo* nucleate actin filaments, similarly to gelsolin (Burtnick et al., 2001; Nag et al., 2013; Kis-Bicskei et al., 2018). To test the nucleation ability of Fli-I the number of actin filaments formed in the absence or presence of Fli-I GST-GH16 was measured at steady-state by TIRFM (Figures 6E,F). For this purpose, actin filaments were allowed to form spontaneously or in the presence of a relatively high concentration of Fli-I GST-GH16 (800 nM) overnight, followed by phalloidin stabilization and dilution. In the absence of Fli-I GST-GH16, the number of actin filaments was found to be *N* = 11.78 ± 5.12, while Fli-I GST-GH16 increased this parameter significantly by ∼4-fold (*N* = 49.06 ± 15.20, *p* ≤ 0.0001) (Figure 6F). On the other hand, the steady-state filament length was markedly reduced in the presence of Fli-I GST-GH16 as compared to the control, further supporting the polymerization inhibitory activity of Fli-I (Figure 6G). Due to the inhibited lengthening of the filaments by Fli-I, the size of some filaments may be under the resolution limit of the microscope, therefore filament number is expected to be underestimated in our experiments. Thus, it appears that when present at relatively high concentrations, Fli-I can promote actin assembly at a moderate level by facilitating the *de novo* formation of nucleation intermediates that is attributed to its relatively low-affinity monomer binding.

### Profilin Supports Barbed End Capping but Interferes With Monomer Binding of Fli-I

**FIGURE 7:**
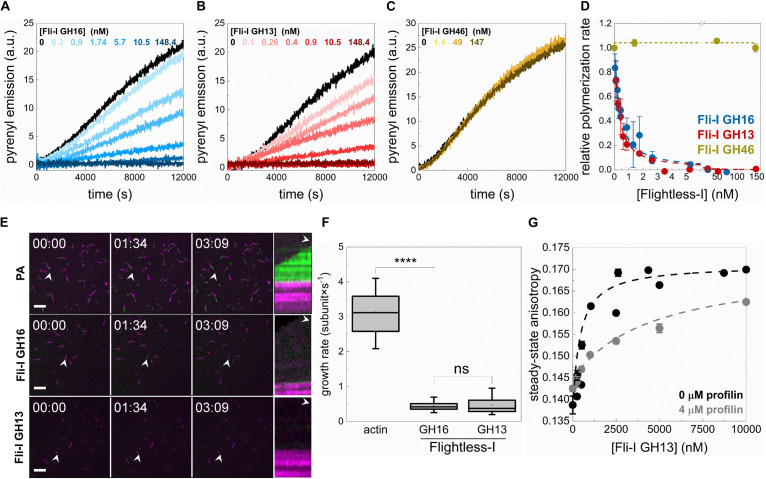
Profilin supports barbed end capping but interferes with the monomer binding activity of Flightless-I. **(A–C)** Polymerization kinetics of profilin:G-actin (PA) followed by the change in pyrenyl emission in the absence or presence of different concentrations of GST-Fli-I. Conditions: [actin] = 2.5 μM (2% pyrenyl labeled), [profilin] = 6 μM. **(D)** Relative polymerization rate as a function of [GST-Fli-I]. Data are shown as mean ± SD, *n* = 2–7. Blue and red dashed lines show the fit to the data (Eq. 1). The fit gave IC_50(Fli–I GST–GH16)_ = 0.93 ± 0.12 nM and IC_50(Fli–I GST–GH13)_ = 0.13 ± 0.01 nM. **(E)** Representative montages of profilin:actin (PA) assembly (green) from preformed F-actin seeds (magenta) followed by TIRFM in the absence or presence of GST-Fli-I. Arrowheads highlight the filaments that were tracked for kymographs. Conditions: [actin] = 0.5 μM (10% Alexa488NHS or Alexa568NHS labeled), [profilin] = 2 μM, [GST-Fli-I] = 10 nM. Scale bar = 10 μm, time = min:s. **(F)** Filament growth rate from profilin:actin in the absence or presence of GST-Fli-I derived from time-lapse TIRFM images shown on **(E)**, *n* = 37–62. **(G)** Steady-state anisotropy of Alexa488NHS-G-actin (0.2 μM) in complex with profilin (4 μM) as a function of [GST-Fli-I]. Data are shown as mean ± SD, *n* = 2–3. Dashed lines in the corresponding color show the fit of the data according to Eq. 3. The fit gave dissociation equilibrium constants of K_D(Fli–I GST–GH13)_ = 411.43 ± 22.69 nM (in the absence of profilin) and K_D(Fli–I GST–GH13)_ = 4511.6 ± 631.92 nM (in the presence of profilin). ****p < 0.0001.

Cellular actin structures are built from profilin:G-actin (PA), therefore we aimed to investigate whether the presence of profilin influences the actin assembly activities of Fli-I. In pyrenyl polymerization experiments, we found that both Fli-I GST-GH16 and GST-GH13 inhibit the assembly of profilin:G-actin at subnanomolar concentrations (Figures 7A–D). However, in contrast to their effects on the assembly of free G-actin, they failed to increase the bulk polymerization rate of profilin:G-actin at higher concentrations. The analysis of the GST-Fli-I concentration dependence of the bulk polymerization rate gave half-inhibitory concentration values of IC_50(Fli–I GST–GH16)_ = 0.93 ± 0.12 nM and IC_50(Fli–I GST–GH13)_ = 0.13 ± 0.01 nM (Figure 7D, Eq. 1). These data indicate that Fli-I prevents the assembly of profilin:G-actin with high-affinity barbed end capping. Similar to the lack of the effect of Fli-I GST-GH46 on actin assembly from free G-actin, this construct failed to influence the polymerization of profilin:G-actin (Figures 7C,D).

Dual-color TIRFM experiments performed to study profilin:G-actin assembly corroborated our observations made in spectroscopic assays (Figures 7E,F). Polymer growth (Figure 7E, green portion) was observed from preformed F-actin seeds (Figure 7E, magenta portion) both in the absence and presence of Fli-I. In control samples, profilin:G-actin assembled at the barbed ends of preformed F-actin actin seeds at a rate of v_*PA*_ = 3.14 ± 0.58 subunit × s^–1^ (*n* = 37) that is consistent with the slightly reduced association rate constant of profilin:G-actin to the barbed ends as compared to free G-actin (k_+_ = 7.86 ± 1.45 subunit × s^–1^; Barko et al., 2010; Toth et al., 2016; Vig et al., 2017; Figure 7F). In the presence of Fli-I GST-GH16 (10 nM) or GST-GH13 (10 nM) the number of elongating barbed ends, as well as the rate of profilin:G-actin association to F-actin seeds was severely reduced [v_Fli–I GST–GH16_ = 0.45 ± 0.14 subunit × s^–1^ (*n* = 62, *p* ≤ 0.0001), v_Fli–I GST–GH13_ = 0.46 ± 0.23 subunit × s^–1^ (*n* = 57)] (Figures 7E,F).

The lack of the polymerization promoting effect that we detect in the presence of profilin indicates that the interaction of Fli-I with monomeric actin is modulated by profilin. To test this, the steady-state anisotropy of fluorescently labeled actin (0.2 μM Alexa488NHS-G-actin) in complex with profilin (4 μM) was measured upon titration with Fli-I GST-GH13 (Figure 7G). The analysis revealed that the binding strength of Fli-I GST-GH13 to profilin:G-actin is markedly reduced (K_D(Fli–I GST–GH13)_ > 4 μM) as compared to that of free G-actin (K_D(Fli–I GST–GH13)_ ∼500–600 nM, Figures 3B,C, 7G). These observations are consistent with the ability of profilin to inhibit Fli-I:G-actin interaction and suggest that Fli-I and profilin bind to monomeric actin in a competitive fashion.

### The Influence of the GST Fusion on the Actin Activities of Fli-I

**FIGURE 8:**
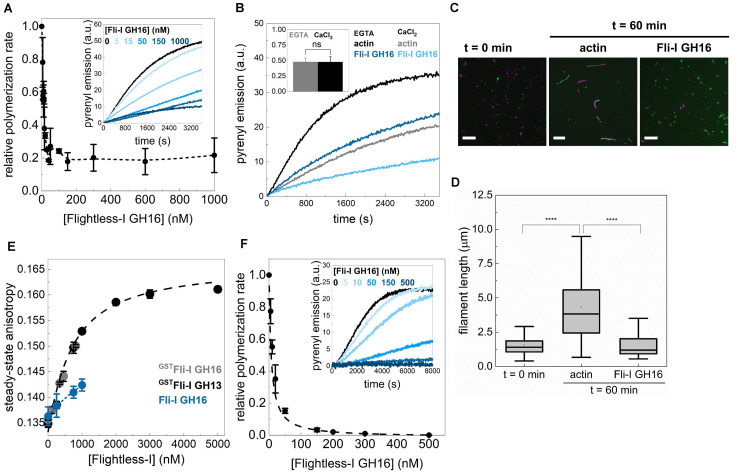
The influence of the GST fusion on the actin activities of Flightless-I. **(A)** Relative polymerization rate as a function of [Fli-I GH16]. Data are shown as mean ± SD, *n* = 2–7. Inset: representative pyrenyl fluorescence emission kinetics recorded in the absence or presence of different concentrations of Fli-I GH16. Conditions: 2.5 μM actin (5% pyrenyl labeled). **(B)** Representative pyrenyl emission kinetics recorded in the absence or presence of Fli-I GH16 (14 nM) and in the presence of 1 mM EGTA or 1 mM CaCl_2_. Conditions: 2.5 μM actin (5% pyrenyl labeled). Inset: Relative polymerization rates derived from pyrenyl transients. Data are shown as mean ± SD, *n* = 2–4. **(C)** Representative montage of actin filament end-to-end annealing followed by TIRFM in the absence or presence of Fli-I GH16. The *t* = 0 min corresponds to the initial fragmentation of filaments, while t = 60 min indicates the time interval after fragmentation. Conditions: [actin] = 0.5 μM (10% Alexa488NHS and 10% Alexa568NHS labeled), [Fli-I GH16] = 100 nM. Scale bar = 10 μm. **(D)** Filament length at *t* = 0 min and 60 min in the absence or presence of Fli-I GH16, derived from TIRFM images shown on **(C)**, n_*samples*_ = 5–10, n_Filaments_ = 100–292. **(E)** Steady-state anisotropy of Alexa488NHS-G-actin (0.2 μM) as a function of [Fli-I GH16] in the presence of 1 mM EGTA. The datasets shown in black and gray are shown for comparison and the same as presented in Figure 3B. Data are shown as mean ± SD, *n* = 3. **(F)** Relative polymerization rate of profilin:G-actin as a function of [Fli-I GH16]. Data are shown as mean ± SD, *n* = 2–4. Inset: representative pyrenyl fluorescence emission kinetics recorded in the absence or presence of different concentrations of Fli-I GH16. Conditions: 2.5 μM actin (5% pyrenyl labeled), [profilin] = 6 μM. Dashed line shows the fit to the data (Eq. 1). The fit gave IC_50(Fli–I GH16)_ = 6.92 ± 0.03 nM. ****p < 0.0001.

Although previous studies (Goshima et al., 1999; Li et al., 2008; Mohammad et al., 2012), as well as our initial set of experiments, were carried out by using GST-tagged Fli-I proteins, as an additional control, we wanted to check the potential of GST to influence the activities of Fli-I on actin dynamics (Figure 8). We found that Fli-I GH16 lacking the GST-tag inhibits actin assembly kinetics in a calcium-independent fashion, and also the end-to-end annealing of actin filaments at nM concentrations (Figures 8A–D). This is nearly identical to our results with the GST fusion construct, and therefore these observations corroborate the high-affinity barbed end capping of the native gelsolin homology domains of Fli-I. However, higher concentrations of the tag-free Fli-I GH16 (∼1 μM, the maximum amount that could be tested in the experiments) only modestly increased the rate of pyrenyl actin assembly (Figure 8A, inset dark blue curve), which is weaker than the apparent polymerization promoting effect observed for Fli-I GST-GH16 (Figures 4A,E). Although no complete assembly inhibition was observed, the polymerization rate was decreased to ∼20% by the cleaved construct (Figure 8A). A similar residual ∼20% polymerization activity was detected in the interim regime (∼5–10 nM) in the presence of Fli-I GST-GH16 (Figure 4E, *inset*). To test for G-actin interaction directly, we performed steady-state anisotropy measurements and revealed that, similar to Fli-I GST-GH16, Fli-I GH16 can bind to G-actin, but the removal of the GST-tag attenuates the interaction (Figure 8E). Due to the limitation of applying higher protein concentrations, we could not record enough data points for reliable quantitative analysis. Nevertheless, we estimate that the interaction is characterized by an affinity in the few μM range. We also found that the tag-free Fli-I GH16 inhibits completely the assembly of profilin:G-actin (Figure 8F). The half-inhibitory concentration was found to be IC_50(Fli–I GH16)_ = 6.92 ± 0.03 nM (Figure 8F, Eq. 1). This value is somewhat larger than that of Fli-I GST-GH16; still, it is important to emphasize that it reflects a high-affinity barbed end interaction.

These results indicate that caution is highly recommended when interpreting data gathered with GST-tagged proteins. Whereas no qualitative difference was found, it appears that the quantitative nature of the *in vitro* G-actin and filament end interactions of Fli-I are influenced by the GST tag. This effect might be explained by GST-mediated stabilization of the protein structure or GST-facilitated dimerization (Gould et al., 2011; Bell et al., 2013; Zhao et al., 2013). Nonetheless, based on the above comparison of the *in vitro* properties of the GST-tagged versus GST-cleaved versions of Fli-I GH16 we conclude that the calcium-independent high-affinity barbed end capping activity and the lower-affinity monomer binding ability of the gelsolin homology domains are retained in the native, GST cleaved state, and for this reason, our major conclusions do not require modifications.

### The GH46 Domains of Fli-I Interact With the C-Terminus of DAAM and Negatively Regulate Its Actin Assembly Activities

**FIGURE 9:**
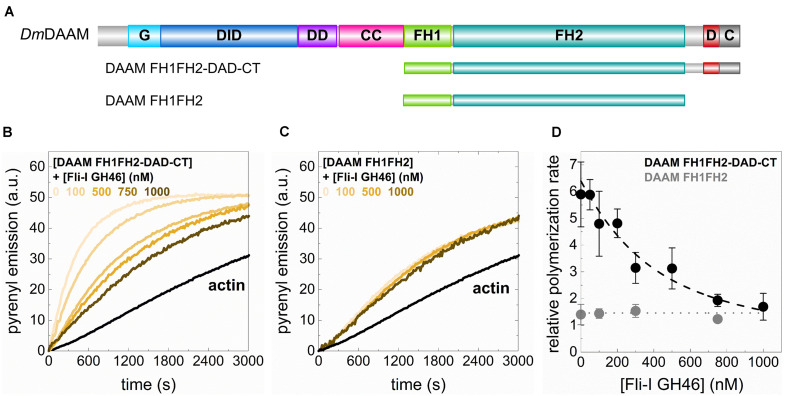
The GH46 domains of Flightless-I interact with the C-terminus of DAAM and negatively regulate its actin assembly activities. **(A)** Domain organization of the DAAM constructs used in our study (DAAM FH1FH2: 552–1054 aa, DAAM FH1FH2-DAD-CT: 552–1153 aa). G, GTPase binding domain; DID, Diaphanous inhibitory domain; DD, dimerization domain; CC, coiled-coil; FH, formin homology domain; D, Diaphanous autoregulatory domain; C, C-terminus. **(B,C)** Representative pyrenyl emission kinetics recorded in the absence or presence of DAAM FH1FH2-DAD-CT or DAAM FH1FH2 at different concentrations of Fli-I GST-GH46. Conditions: 2.5 μM actin (5% pyrenyl labeled), [DAAM] = 250 nM. **(D)** Relative polymerization rates as a function of [Fli-I GST-GH46]. Black dashed line shows the fit of the data according to Eq. 2. The fit gave dissociation equilibrium constant of K_D_ = 255 ± 189 nM.

In the above experiments, we did not detect any direct actin interactions or activities of the GH46 domains of Fli-I. Previously, the GH46 region of the human Fli-I protein was shown to interact with the C-terminal Diaphanous autoinhibitory domain (DAD) of human mDia1 and Disheveled-associated activator of morphogenesis (Daam) 1 formins *in vitro*, and proposed to enhance their actin assembly promoting activities (Higashi et al., 2010). The interaction was found to be specific to Fli-I since the binding was not detected for the GH46 domains of gelsolin. Based on this we sought to investigate the effects of Fli-I GST-GH46 on *Drosophila* DAAM catalyzed actin assembly. An N-terminally truncated, constitutively active DAAM construct comprising the formin homology (FH) domains, FH1 and FH2 and the C-terminal DAD-CT regions (FH1FH2-DAD-CT), as well as the isolated DAAM FH1FH2 domains, were studied in pyrenyl polymerization experiments (Figure 9A; Matusek et al., 2008; Barko et al., 2010; Vig et al., 2017). Our previous work showed that the FH1FH2-DAD-CT of DAAM is more potent in promoting actin assembly as compared to FH1FH2 due to the presence of the DAD-CT region (Vig et al., 2017; Figures 9B,C). We found that while Fli-I GST-GH46 does not influence FH1FH2-mediated actin polymerization, it inhibits the DAAM FH1FH2-DAD-CT-catalyzed actin assembly in a concentration-dependent fashion (Figures 9B,C). At maximum saturation, the assembly rate corresponded to that of characteristic to DAAM FH1FH2 (Figure 9D). Analysis of the data gave the dissociation equilibrium constant of the Fli-I GST-GH46:DAAM interaction of K_D_ = 255 ± 189 nM (Eq. 2).

These results indicate that Fli-I GST-GH46 binds to DAAM through the DAD-CT region, which is in agreement with previous findings (Higashi et al., 2010) and reveal that the interaction is conserved from fruitfly to human. On the other hand, our data suggest that the binding of Fli-I to DAAM inhibits the contribution of DAD-CT to the actin assembly promoting activity of FH1FH2 (Vig et al., 2017) and thereby negatively regulates the effect of DAAM on actin polymerization.

### Fli-I GH13 Disrupts the Actin Cytoskeleton *in vivo*

**FIGURE 10:**
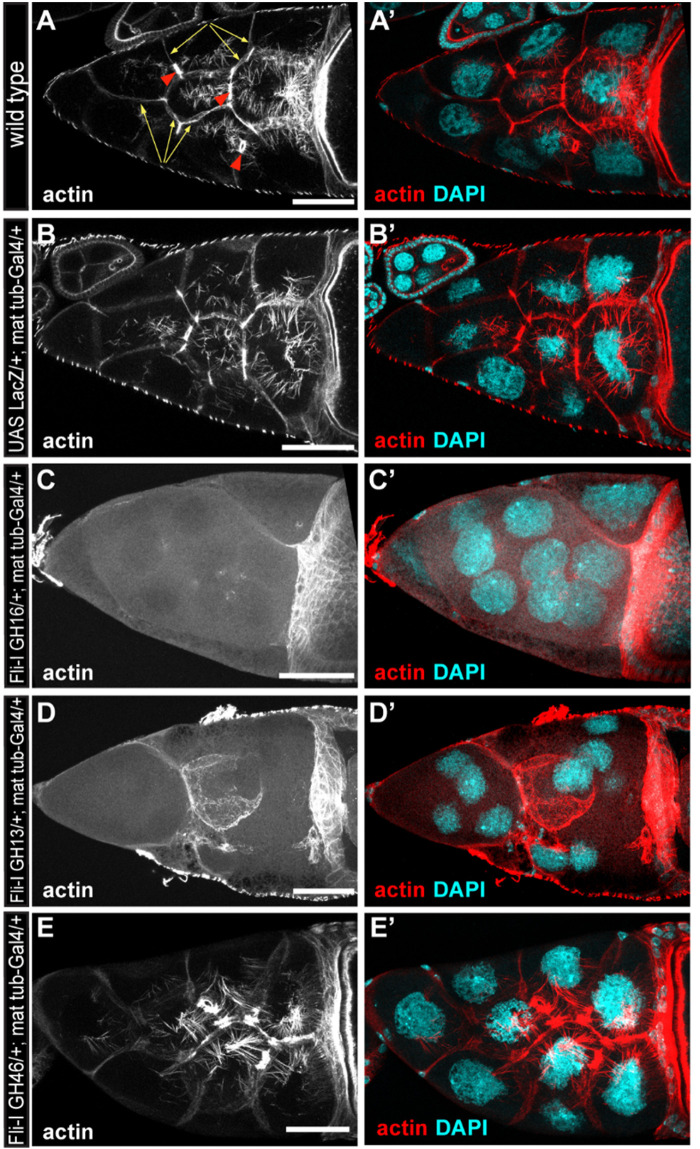
The effect of the gelsolin homology domains of Fli-I on actin organization in *Drosophila* egg chambers. **(A,A’)** Actin organization in nurse cells of a wild type *Drosophila* egg chamber in stage 10B is characterized by the presence of cortical actin (yellow arrows), ring canals (red arrowheads) and cytoplasmic actin cables growing from the plasma membrane to the nucleus. Actin is shown in **(A)** in grayscale, an overlay of DAPI (in cyan) and actin (in red) are shown in **(A’)**. **(B,B’)** Maternal expression of a UAS-lacZ control does not affect actin organization in the nurse cells. **(C–D’)** Maternal expression of UAS-Fli-I GH16 **(C,C’)** or UAS-Fli-I GH13 **(D,D’)** severely impairs cytokinesis in the nurse cells resulting in fused cells with reduced cortical actin level, ring canals are not evident and the nuclear positioning cytoplasmic actin cables are also missing. **(E,E’)** By contrast, in UAS-Fli-I GH46 expressing egg chambers actin organization is not altered as compared to wild type. Anterior edge of the oocyte is visible on the right side of each panel; anterior is on the left, posterior is on the right. Scale bar = 50 μm.

To assess the *in vivo* significance of our *in vitro* findings, we tested the effect of Fli-I overexpression in developing *Drosophila* egg chambers. A wild type egg chamber is composed of 16 germ cells (the oocyte and 15 nurse cells) surrounded by the somatic follicle cells forming a single cell layer around the oocyte and the nurse cells (Figures 10A,A’). Stage 10B egg chambers contain various types of actin-rich structures such as a prominent cortical actin network, ring canals and a nuclear positioning stress fiber-like system in the nurse cells (Figures 10A,A’). Because Fli-I is not known to contribute to the formation of these actin structures (Perrimon et al., 1989; Campbell et al., 1993), the egg chamber appeared as a suitable model system to study the consequences of ectopic expression of Fli-I in an *in vivo* system. To this end, we created transgenic lines for Fli-I GH16, GH13 and GH46 under UAS control and expressed the proteins with *mat-tub4-Gal4* in the germline cells of the ovary. Overexpression of a UAS-LacZ control line had no effect on actin organization of the egg chambers (*n* = 45) (Figures 10B,B’), whereas that of GH16 (*n* = 39) and GH13 (*n* = 42) caused a severe disruption of the cortical actin network of the nurse cells in every egg chamber examined, often resulting in giant nurse cells with multiple nuclei due to fusion of their cytoplasm (Figures 10C–D’). In addition, we observed markedly reduced actin accumulation around the ring canals and largely reduced levels of the nuclear positioning actin cables (Figures 10C–D’). By contrast, expression of GH46 did not influence actin organization in the nurse cells or the oocyte (*n* = 43) (Figures 10E,E’). These observations suggest that the presence of GH13 interferes with the formation of various types of actin cables, while GH46 has no such an effect. This effect of GH13 is entirely consistent with the *in vitro* barbed end capping activity of GH13 that provides a plausible molecular mechanism for the *in vivo* effect. Thus, we conclude that our *in vitro* and *in vivo* data both support that of the truncated Fli-I GH domains GH13 is a potent inhibitor of actin polymerization whereas GH46 does not contribute to actin interaction.

## Discussion

We have analyzed the activities of *Drosophila* Fli-I in actin dynamics *in vitro* by using a combination of bulk fluorescence and individual filament approaches. We found that the gelsolin homology domains of Fli-I possess calcium-independent activities in the regulation of actin dynamics, in agreement with previous reports (Figure 3; Goshima et al., 1999; Mohammad et al., 2012). The experimental data agree well with the bioinformatics analysis that predicts the lack of the conservation of structural elements in Fli-I essential for the calcium-dependent regulation of gelsolin (Goshima et al., 1999; Mohammad et al., 2012; Nag et al., 2013; Figure 2). The calcium insensitivity of the Fli-I:actin interaction suggests that the gelsolin homology domains of Fli-I adopt a conformation different from that of gelsolin, in which the actin-binding interface is constitutively exposed.

We showed that gelsolin homology domains of Fli-I interact directly with both actin filaments and monomers (Figures 3–6; Liu and Yin, 1998; Goshima et al., 1999; Mohammad et al., 2012). Importantly, our work reveals that the F-actin and G-actin binding of Fli-I is characterized by markedly different affinities in the ∼nM and ∼μM range, respectively. The magnitude of the actin affinities of Fli-I agrees well with the preferential association of the protein to F-actin against G-actin detected in 3T3 and 293T cell lysates (Li et al., 2008; Mohammad et al., 2012). The binding strength but not the qualitative nature of the interaction between Fli-I GH16 and actin is influenced by the GST fusion of the protein (Figure 8). This indicates that GST may influence the folding of the protein and/or promote the ability of Fli-I to dimerize (Gould et al., 2011; Bell et al., 2013; Zhao et al., 2013). Our work identifies the GH13 domains as the main actin interacting region of Fli-I, since neither the C-terminal GH46 domains nor the leucine-rich repeat region at the N terminus associates to actin (Figures 3–6). Bioinformatics analysis predicts that residues responsible for gelsolin:actin interactions show a rather weak similarity in Fli-I, with the highest conservation in the GH1 domain (Figure 2). In agreement with our observations, previous investigations found that the GH1 truncated human Fli-I, GH26 did not influence the level of steady-state pyrenyl fluorescence suggesting the critical importance of GH1 in actin interaction (Arora et al., 2015). Also, the human Fli-I protein carrying the E586K mutation in the GH1 domain failed to coimmunoprecipitate with actin in 293T cells (He et al., 2018). Based on these considerations the GH1 domain of Fli-I is likely to be the main actin-binding site of the protein.

Our work suggests that the multifunctional nature of gelsolin is not characteristic of Fli-I. The analysis of the activities of Fli-I at the level of individual filaments demonstrates that, as a functional consequence of the high-affinity actin interaction, Fli-GH16 inhibits actin filament elongation by efficiently capping filament barbed ends (Figure 5). Although *C. elegans* Fli-I was proposed to possess filament severing activity based on the appearance of short actin oligomers on electron microscopy images (Goshima et al., 1999), we have not found any evidence for an actin filament severing by Fli-I (Figure 6). As an alternative explanation barbed end capping and limited filament elongation would also result in short actin filaments as we observed in TIRFM experiments (Figures 5, 6). Severing by gelsolin requires the concerted targeting of both its GH1 and GH4 domains to subunit:subunit interfaces at the opposite sides of the actin filament that imposes simultaneous steric clashes competing off the intersubunit interactions (Nag et al., 2013). Besides, the type 1 calcium binding sites of gelsolin may also contribute to severing by disrupting the intersubunit cation binding site (Glu167) of actin (Nag et al., 2013). From this aspect, the lack of actin binding by GH46 and conservation of the type 1 calcium binding sites in Fli-I already predicts that this protein is very unlikely to possess a severing activity, and this is entirely confirmed by our experimental results. We observed that Fli-I can moderately facilitate the formation of nucleation intermediates due to its low-affinity binding to monomeric actin (Figure 6). Importantly, profilin interferes with the monomer binding of Fli-I; therefore in the presence of profilin Fli-I is only able to cap actin filament barbed ends but fails to promote actin assembly from profilin:actin (Figure 7). The negative influence of profilin on the Fli-I:G-actin interaction indicates that the actin-binding sites of profilin and Fli-I are likely to overlap. High-resolution structural analysis showed that the GH1 of gelsolin binds in the barbed end groove of actin subdomains 1 and 3 at a site that is shared by profilin (Schutt et al., 1993; Burtnick et al., 2004; Nag et al., 2009). Hence, these data suggest that the GH1 region of Fli-I adopts a similar binding mode to actin as gelsolin. Collectively, our biochemical analyses indicate that the most prominent actin interaction of Fli-I is the high-affinity filament end binding that endows the protein with barbed end capping activity. In accordance with this, while GH46 has no effect on *in vivo* actin polymerization, as expected from the excess of a barbed end capping protein, overexpression of GH13 prevents the formation of various types of actin cables in the developing *Drosophila* egg chambers (Figure 10).

Although Fli-I GH46 does not interact with actin, we found that it influences formin-mediated actin assembly *in vitro* (Figure 9). Fli-I GH46 inhibits actin assembly catalyzed by DAAM FH1FH2-DAD-CT but does not affect that of mediated by DAAM FH1FH2. These observations indicate that Fli-I GH46 interacts with the C-terminal DAD-CT region of DAAM, corroborating previous studies (Higashi et al., 2010). Our work reveals that the binding strength of the interaction falls in the submicromolar range. The functional consequence of this interaction remains to be elucidated. DAD is involved in the RhoGTPase-dependent regulation of the activities of DAAM through its association to the N-terminal Diaphanous inhibitory domain (DID) (Liu et al., 2008; Vig et al., 2017). This led to the proposal that the Fli-I:DAAM interaction may interfere with this autoinhibitory mechanism and thereby promotes the activation of DAAM (Higashi et al., 2010). Contrasting to this prediction, in our *in vitro* system Fli-I had an inhibitory effect. DAD-CT is involved in the interaction of DAAM with both actin monomers and filament ends and positively regulates the nucleation and elongation activities of the protein *in vitro* (Vig et al., 2017). This can provide a plausible explanation for our *in vitro* observations. Because *in vitro* in the absence of the DID domain the Fli-I/DAD-CT interaction might be more stable than the presumably transient interaction *in vivo*, further investigations are needed to resolve this issue. Nevertheless, other functional alternatives can also be considered. We have formerly shown that the DAD-CT region is important in microtubule interaction, as well as in the actin filament:microtubule coalignment activity of DAAM (Szikora et al., 2017). Based on this, one can hypothesize that the Fli-I:DAAM interaction has a role in the regulation of the microtubule cytoskeleton or the actin:microtubule cross-talk. In support of this, Fli-I was localized to both actin and microtubule-based structures in Swiss 3T3 fibroblasts (Davy et al., 2001). Thus, besides its activities directly targeting actin, the GH46:DAAM interaction can provide an indirect way for Fli-I to regulate actin dynamics and/or to extend its activities toward the microtubule cytoskeleton through a formin-mediated pathway.

## Materials and Methods

### Protein Expression and Purification

The cDNAs of *Drosophila melanogaster* Flightless-I subfragments were cloned into pGEX-6P1 vector (GH1-6: 461–1256 aa) and into pGEX-2T vector (GH1-3: 461–881, GH4-6: 851–1256, LRR: 1–460). Flightless-I constructs were expressed as glutathione S-transferase (GST) fusion proteins in *Escherichia coli* BL21(DE3)pLysS strain (Novagen). The transformed bacteria were grown at 37°C in Luria Broth (Lennox) EZMix^TM^ powder microbial growth medium (Sigma-Aldrich) overnight. Protein expression was induced with 0.5 mM isopropyl β-D-1-thiogalactopyranoside (IPTG) at OD_600_ ∼0.6–0.8. After overnight expression at 20°C the bacterial pellet was collected by centrifugation (Hermle Z326K; 10,000 g, 10 min, 4°C) and stored at –20°C until use. For protein purification the bacterial pellet was lysed by sonication in Lysis buffer [20 mM Tris-HCl pH 8.0, 1 M NaCl, 5 mM CaCl_2_, 1 mM ATP, 0.5% Triton X-100, 1% sucrose, 2 mM DTT, 5% glycerol supplemented with 0.1 mM PMSF and Protease Inhibitor Cocktail (Sigma-Aldrich, P8465)]. The cell lysate was centrifuged (Hitachi CP 80NX; 10,000 g, 25 min, 4°C) and the supernatant was gently stirred with 0.8% (m/v) polyethyleneimine solution (pH 7.9, PEI, Sigma-Aldrich) on ice to precipitate nucleic acids. Subsequently, the solution was centrifuged (Hitachi CP 80NX; 17,300 g, 10 min, 4°C) and solid, fine powdered ammonium sulfate (AS, Sigma-Aldrich) was added to the supernatant to 60% saturation by gently stirring it for 30–45 min to precipitate the proteins. The precipitate was collected by centrifugation (Hitachi CP 80NX; 21.700 g, 25 min, 4°C). The pellet was resuspended in Lysis buffer and precipitated repeatedly by adding a 60% saturated AS solution to remove the redundant PEI. After centrifugation (Hitachi CP 80NX; 21.700 g, 25 min, 4°C), the pellet was resuspended in Low salt buffer (20 mM TRIS pH 7.9, 50 mM NaCl, 1% sucrose, 5% glycerol, 1 mM DTT). The solution was incubated with Glutathione Sepharose 4B resin (Sigma-Aldrich) at 4°C overnight. Subsequently, it was loaded onto a column and washed with Low salt buffer. The GST-tagged proteins were eluted with 50 mM glutathione dissolved in Low salt buffer (Glutathione Reduced, Sigma-Aldrich). Alternatively, the GST tag from Fli-I G16 was cleaved by PreScission Protease on column (4^o^C, 15 min) and the cleaved protein was collected as flowthrough. Fli-I proteins were concentrated with Amicon-Ultra 50 kDa tube (Merck Millipore) by centrifugation (Hermle Z326K; 3000 g, 5 min, 4°C). The concentrate was loaded onto a PD10 column (GE Healthcare) for buffer exchange into Low salt buffer. Before flash freezing in liquid nitrogen the constructs were clarified by ultracentrifugation (Beckman Coulter; 300,000 g, 30 min, 4°C) and stored at –80°C until use. Control experiments showed that a freeze/thaw cycle does not affect the functionality of the constructs (*data are not shown*). The concentrations were measured spectrophotometrically using the extinction coefficients derived from the amino acid sequence (ExPAsy ProtParam tool)^1^. The purity of the protein was checked by UV-VIS absorption photometry by calculating the A_280_/A_260_ ratio (Manchester, 1995), which was found to be >1.7. We found that such a labor-intensive method was essential for the efficient removal of nucleic acid contamination from recombinantly produced Flightless-I. Of note, that difficulty in isolation of soluble recombinant Flightless-I protein were reported (Liu and Yin, 1998). Rabbit skeletal muscle actin (actin) was purified, gel filtered on Superdex 200 (GE Healthcare) and stored in G-buffer (4 mM TRIS, pH 7.8, 0.1 mM CaCl_2_, 0.2 mM ATP, 0.5 mM DTT) as described previously (Spudich and Watt, 1971; Toth et al., 2016; Vig et al., 2017). The actin bound calcium was replaced to magnesium before the measurements by adding 200 μM EGTA and 50 μM MgCl_2_. Actin was labeled at Lys^328^ by Alexa Fluor^®^ 488 carboxylic acid succinimidyl ester (Alexa488NHS, Invitrogen), Alexa Fluor^®^ 568 carboxylic acid succinimidyl ester (Alexa568NHS, Invitrogen) or at Cys^374^ by N-(1-pyrene)iodoacetamide (pyrene, Thermo Fisher Scientific) according to standard protocols (Toth et al., 2016; Vig et al., 2017). Mouse profilin 1 (profilin), human gelsolin (GSN) and fragments of *Drosophila* Disheveled-associated activator of morphogenesis (DAAM) formin were obtained as described previously (Toth et al., 2016; Szikora et al., 2017; Vig et al., 2017).

### Fluorescence Spectroscopy

#### Bulk Actin Assembly/Disassembly Measurements

Pyrene-actin assembly assay was performed using gel-filtered actin (2.5 μM, containing 2 or 5% pyrene labeled actin in the presence and absence of profilin, respectively). The polymerization of magnesium-actin was initiated by the addition of 1 mM MgCl_2_ and 50 mM KCl in the absence and presence of different concentrations of Fli-I proteins. To address the effect of Ca^2+^ on the actin activities of Fli-I or GSN measurements were performed either in the presence of 1 mM EGTA (Ca^2+^-free condition) or 1 mM CaCl_2_. In measurements when profilin was present the profilin concentration was 6 μM. Considering the dissociation equilibrium constant of profilin:G-actin to be K_D_ ∼0.2 μM, ∼95% of the monomeric actin was bound to profilin under these experimental conditions. Pyrene fluorescence emission was monitored by a Safas Xenius FLX spectrofluorimeter (λ_ex_ = 365 nm, λ_em_ = 407 nm). For quantitative analysis, the *polymerization rate* was derived as the slope of the linear part of the pyrene trace at each condition. The relative polymerization rate was calculated as the ratio of polymerization rates obtained in the presence and absence of Fli-I proteins. The relative polymerization rate (*v*_relative_) of profilin:actin as a function of [Fli-I] (*F*_0_) was fit by the following equation:

(1) $$v_{relative}=1-\frac{1-\frac{v_{min}}{v_{0}}}{1+\frac{IC_{50}}{F_{0}}}$$

where *v*_0_ and *v*_min_ are the relative polymerization rates in the absence and presence of saturating amount of Fli-I, respectively and *IC*_*50*_ is the Fli-I concentration corresponding to 50% inhibition.

The Fli-I dependence of the rate of DAAM FH1FH2-DAD-CT catalyzed actin assembly (*v*) was fit by the following equation:

(2) $$\frac{v-v_{max}}{v_{min}-v_{max}}=\frac{A_{0}+F_{0}+K_{D}-\sqrt{{\left(A_{0}+F_{0}+K_{D}\right)}^{2}-4A_{0}F_{0}}}{2F_{0}}$$

where *v*_min_ and *v*_max_ are the polymerization rates in the absence and presence of saturating amount of [Fli-I]; *A*_0_ and *F*_0_ are the total DAAM FH1FH2-DAD-CT and Fli-I concentration, respectively, *K*_D_ is the dissociation equilibrium constant of the DAAM:Fli-I complex.

#### Steady-State Anisotropy Measurements

To study the Fli-I:G-actin interaction the steady-state anisotropy (anisotropy) of Alexa488NHS-Mg^2+^-ATP-G-actin (Alexa488NHS-G-actin) was measured. Alexa488NHS-G-actin (0.2 μM) was incubated with Latrunculin A (LatA, 4 μM) for 15 min at room temperature. Then Fli-I constructs were added at different concentrations and the samples were further incubated for 1 h at 22^o^C either in the presence of 1 mM EGTA (Ca^2+^-free condition) or 1 mM CaCl_2_. In measurements when profilin was present profilin (4 μM) was added to actin after the incubation with LatA and the samples were further incubated for 1 h at 22^o^C before the addition of Fli-I constructs. Similarly, to the kinetic analysis, the anisotropy measurements were performed in the presence of 1 mM MgCl_2_ and 50 mM KCl/NaCl. Since the presence of LatA prevents actin polymerization, thus the increase in steady-state anisotropy could not result from filament formation, it solely reflects the binding of Fli-I to actin. Anisotropy was measured using a Horiba Jobin Yvon Fluorolog-3 spectrofluorometer (λ_ex_ = 488 nm, λ_em_ = 516 nm) excitation and emission slits were both set to 5 nm. For quantitative analysis the Fli-I concentration dependence of the steady-state anisotropy (*r*) measured either in the absence or presence of profilin was fit by the following equation:

(3) $$\frac{r-r_{\text{A}}}{r_{\text{AF}}-r_{\text{A}}}=\frac{A_{0}+F_{0}+K_{\text{D}}-\sqrt{{\left(A_{0}+F_{0}+K_{\text{D}}\right)}^{2}-4A_{0}F_{0}}}{2F_{0}}$$

where *A*_0_ and *F*_0_ are the total G-actin and Fli-I concentration, respectively, *r*_A_ is the steady-state anisotropy of Alexa488NHS-G-actin, *r*_AF_ is the steady-state anisotropy of Alexa488NHS-G-actin at saturating amount of Fli-I and *K*_D_ is the dissociation equilibrium constant of the G-actin:Fli-I complex.

### Total Internal Reflection Fluorescence Microscopy

#### Actin Assembly/Disassembly Measurements

The effects of Fli-I on the assembly/disassembly of individual actin filaments were studied by total internal reflection fluorescence microscopy (TIRFM). Glass flow cells were incubated with 1 volume of N-ethylmaleimide myosin for 1 min, washed extensively with 2 volumes of myosin buffer (F buffer supplemented with 0.5 M KCl; F buffer = G buffer supplemented with 1 mM MgCl_2_ and 50 mM KCl) and 1 volume of 1% (m/v) BSA (dissolved in F buffer) and equilibrated with 2 volumes of TIRF buffer (0.5% (m/v) methylcellulose, 0.5% (m/v) BSA, 10 mM 1,4-diazabicyclo[2,2,2]octane (DABCO), 100 mM DTT dissolved in F buffer). The polymerization of magnesium-actin (0.5 μM, 10% Alexa488NHS labeled) was initiated by the addition of 1 mM MgCl_2_ and 50 mM KCl in the absence or presence of different concentrations of Fli-I constructs. Dual-color TIRF experiments were performed to follow the assembly of G-actin in the absence or presence of Fli-I and profilin. G-actin (0.5 μM, 10% Alexa568NHS labeled) was polymerized in the flow cell for 10 min to form “magenta” actin filaments, then unpolymerized actin was washed out by 1 volume of TIRF buffer. A mixture of G-actin (0.5 μM, 10% Alexa488NHS labeled), profilin (2 μM) and Fli-I in TIRF buffer was transferred into the flow cell. Time-lapse images of actin assembly were captured every 10.5 s with an Olympus IX81 microscope (laser lines: 491 nm and 561 nm, APON TIRF 60x NA1.45 oil immersion objective, Hamamatsu CCD camera). In assembly assays, to derive the elongation rate of actin filaments time-lapse images were analyzed by either the MultipleKymograph plugin of Fiji or by manually measuring filament lengthening. The elongation rate of actin filaments (*v*) was related to the critical concentration of actin assembly (*c*_c_ ∼ 0.1 μM; Pollard, 2007; Bugyi and Carlier, 2010), to the association rate constant of actin monomers to filament barbed ends (*k*_*+*_) and to the total actin concentration ([*G*_0_]) by the following equation:

(4) $$v=k_{+}\left(\left[G_{0}\right]-c_{c}\right)$$

In disassembly assays actin (0.5 μM, 10% Alexa488NHS labeled) was allowed to polymerize for 35 min in the flow cell, then buffer conditions were changed to TIRF buffer supplemented with Fli-I (105 nM) or gelsolin (5 nM). Time-lapse images of actin disassembly were captured every 10.5 s with an Olympus IX81 microscope (laser lines: 491 and 561 nm, APON TIRF 60x NA1.45 oil immersion objective, Hamamatsu CCD camera). The percent area covered by actin filaments 5 min after the initiation of actin disassembly was derived from a 66 × 66 μm^2^ region of the images by using Fiji.

#### Annealing Experiments

To study the end-to-end annealing of actin filaments (1 μM, containing either 10% Alexa488NHS or 10% Alexa568NHS labeled actin) were allowed to polymerize 1 h at room temperature and stabilized by phalloidin (1:1 molar ratio) overnight. Filaments with different spectral properties were mixed either in the absence or presence of Fli-I and subsequently fragmented by a 26G syringe (10×). Samples were diluted to 2 nM actin by TIRF buffer and were processed for microscopy analysis. Images were captured immediately after fragmentation (*t* = 0 min) and after 60 min (*t* = 60 min) with an Olympus IX81 microscope (laser lines: 491 and 561 nm, APON TIRF 60x NA1.45 oil immersion objective, Hamamatsu CCD camera). The length of the actin filaments was derived by using Fiji.

#### Steady-State Measurements of Actin Filament Number

Actin filaments (2 μM) were allowed to polymerize overnight either in the absence or presence of Fli-I GH16 (800 nM). Filaments were stabilized by AlexaFluor^TM^ 488 Phalloidin (Thermo Fischer Scientific; 1:1 molar ratio), diluted to 5 nM into TIRF buffer and were processed for microscopy analysis. Images were captured with an Olympus IX81 microscope (laser line: 491 nm, APON TIRF 60x NA1.45 oil immersion objective, Hamamatsu CCD camera). The number and length of actin filaments were derived from a 66 × 66 μm^2^ region of the images by using Fiji.

### Fly Work and Immunohistochemistry

Fly-strains were raised under standard laboratory conditions at 25°C. As wild type, we used an isogenized *w*^1118^ stock. For germline expression of the UAS transgenes, we used *mat*α*4 Tub-Gal4* (BDSC#7063). Adult ovaries were dissected in cold PBS, fixed in 4% paraformaldehyde (diluted in PBS) at room temperature for 20 min. After fixation, the samples were washed three times for 20 min in PBS containing 0.1% TritonX-100 (PBST) and blocked in PBST with 1% BSA, 0.02% NaN_3_ and 5% FBS (PBT-N) for 2 h. The antibodies were diluted in PBT-N and incubated overnight at 4°C. Actin was labeled with AlexaFluor^TM^ 546 Phalloidin (Thermo Fischer Scientific, 1:100), nurse cell nuclei were labeled with DAPI (Sigma-Aldrich, 1:500). Samples were mounted in ProLong Gold (Thermo Fisher Scientific). Each staining was repeated twice, and we examined 10–15 egg chambers from each. Confocal images were captured on a Zeiss LSM 800 confocal microscope. Images were edited with ImageJ/Fiji software and Adobe Illustrator CS6.

### Statistical Analysis

The data presented were derived from at least two independent experiments. Values are displayed as mean ± SD. The number of independent experiments is given in the figure legends. Statistical analysis (Student’s *t*-test) was performed by Microsoft Excel (^*ns*^*p* ≥ 0.05, ^∗^*p* < 0.05, ^∗∗^*p* < 0.01, ^∗∗∗^*p* < 0.001, ^****^p < 0.0001). The significance levels are given in the text and on the corresponding figures.

## Data Availability Statement

All datasets presented in this study are included in the article/supplementary material.

## Author Contributions

JM and BB: conceptualization, supervision, and writing – review and editing. GG-G, RP, PB, PG, and BB: formal analysis and visualization. JM, BB, and TH: funding acquisition. GG-G, DF, EM, RP, TH, PB, PG, AV, MT, and BB: investigation. JM, BB, and RP: writing – original draft. All authors contributed to the article and approved the submitted version.

## Conflict of Interest

The authors declare that the research was conducted in the absence of any commercial or financial relationships that could be construed as a potential conflict of interest.

## Footnotes

1 http://web.expasy.org/protparam/

## Abbreviations

C: C-terminus
CapG: macrophage capping protein
CC: coiled-coil
D: Diaphanous autoregulatory domain
DAAM: Disheveled-associated activator of morphogenesis
DD: dimerization domain
DID: Diaphanous inhibitory domain
FH: formin homology domain
Fli-I: Flightless-I
G: GTPase binding domain
GH: gelsolin homology domain
GSN: gelsolin
Lmod: Leiomodin
LRR: leucine-rich repeat
SALS-WH2: Wiskott-Aldrich Syndrome Homology 2 domains of Sarcomere Length Short
TIRFM: total internal reflection fluorescence microscopy.

## References

[B1] Arora P. D. Wang Y. Bresnick A. Janmey P. A. McCulloch C. A. (2015). Flightless I interacts with NMMIIA to promote cell extension formation, which enables collagen remodeling. *Mol. Biol. Cell* 26 2279–2297. 10.1091/mbc.E14-11-1536 25877872 PMC4462945

[B2] Barko S. Bugyi B. Carlier M. F. Gombos R. Matusek T. Mihaly J. (2010). Characterization of the biochemical properties and biological function of the formin homology domains of Drosophila DAAM. *J. Biol. Chem.* 285 13154–13169. 10.1074/jbc.m109.093914 20177055 PMC2857102

[B3] Bell M. R. Engleka M. J. Malik A. Strickler J. E. (2013). To fuse or not to fuse: what is your purpose? *Protein Sci.* 22 1466–1477. 10.1002/pro.2356 24038604 PMC3831663

[B4] Bugyi B. Carlier M. F. (2010). Control of actin filament treadmilling in cell motility. *Annu. Rev. Biophys.* 39 449–470. 10.1146/annurev-biophys-051309-103849 20192778

[B5] Burtnick L. D. Robinson R. C. Choe S. (2001). “Structure and Function of Gelsolin,” in *Molecular Interactions of Actin: Actin Structure and Actin-Binding Proteins*, eds dos Remedios C. G. Thomas D. D. (Berlin: Springer Berlin Heidelberg), 201–211.

[B6] Burtnick L. D. Urosev D. Irobi E. Narayan K. Robinson R. C. (2004). Structure of the N-terminal half of gelsolin bound to actin: roles in severing, apoptosis and FAF. *EMBO J.* 23 2713–2722. 10.1038/sj.emboj.7600280 15215896 PMC514944

[B7] Cameron A. M. Turner C. T. Adams D. H. Jackson J. E. Melville E. Arkell R. M. (2016). Flightless I is a key regulator of the fibroproliferative process in hypertrophic scarring and a target for a novel antiscarring therapy. *Br. J. Dermatol.* 174 786–794. 10.1111/bjd.14263 26521845

[B8] Campbell H. D. Fountain S. McLennan I. S. Berven L. A. Crouch M. F. Davy D. A. (2002). Fliih, a gelsolin-related cytoskeletal regulator essential for early mammalian embryonic development. *Mol. Cell. Biol.* 22 3518–3526. 10.1128/mcb.22.10.3518-3526.2002 11971982 PMC133791

[B9] Campbell H. D. Fountain S. Young I. G. Claudianos C. Hoheisel J. D. Chen K. S. (1997). Genomic structure, evolution, and expression of human FLII, a gelsolin and leucine-rich-repeat family member: overlap with LLGL. *Genomics* 42 46–54. 10.1006/geno.1997.4709 9177775

[B10] Campbell H. D. Schimansky T. Claudianos C. Ozsarac N. Kasprzak A. B. Cotsell J. N. (1993). The Drosophila melanogaster flightless-I gene involved in gastrulation and muscle degeneration encodes gelsolin-like and leucine-rich repeat domains and is conserved in Caenorhabditis elegans and humans. *Proc. Natl. Acad. Sci. U.S.A.* 90:11386. 10.1073/pnas.90.23.11386 8248259 PMC47987

[B11] Chen K. S. Gunaratne P. H. Hoheisel J. D. Young I. G. Miklos G. L. Greenberg F. (1995). The human homologue of the Drosophila melanogaster flightless-I gene (flil) maps within the Smith-Magenis microdeletion critical region in 17p11.2. *Am. J. Hum. Genet.* 56 175–182. 7825574 PMC1801336

[B12] Coue M. Korn E. D. (1985). Interaction of plasma gelsolin with G-actin and F-actin in the presence and absence of calcium ions. *J. Biol. Chem.* 260 15033–15041. 2999102

[B13] Cowin A. J. Adams D. H. Strudwick X. L. Chan H. Hooper J. A. Sander G. R. (2007). Flightless I deficiency enhances wound repair by increasing cell migration and proliferation. *J. Pathol.* 211 572–581. 10.1002/path.2143 17326236

[B14] Davy D. A. Ball E. E. Matthaei K. I. Campbell H. D. Crouch M. F. (2000). The flightless I protein localizes to actin-based structures during embryonic development. *Immunol. Cell Biol.* 78 423–429. 10.1046/j.1440-1711.2000.00926.x 10947868

[B15] Davy D. A. Campbell H. D. Fountain S. de Jong D. Crouch M. F. (2001). The flightless I protein colocalizes with actin- and microtubule-based structures in motile Swiss 3T3 fibroblasts: evidence for the involvement of PI 3-kinase and Ras-related small GTPases. *J. Cell Sci.* 114(Pt 3), 549–562. 11171324 10.1242/jcs.114.3.549

[B16] Deng H. Xia D. Fang B. Zhang H. (2007). The Flightless I homolog, fli-1, regulates anterior/posterior polarity, asymmetric cell division and ovulation during Caenorhabditis elegans development. *Genetics* 177 847–860. 10.1534/genetics.107.078964 17720906 PMC2034648

[B17] Feldt J. Schicht M. Garreis F. Welss J. Schneider U. W. Paulsen F. (2019). Structure, regulation and related diseases of the actin-binding protein gelsolin. *Expert Rev. Mol. Med.* 20:e7. 10.1017/erm.2018.7 30698126

[B18] Ferrary E. Cohen-Tannoudji M. Pehau-Arnaudet G. Lapillonne A. Athman R. Ruiz T. (1999). In vivo, villin is required for Ca(2+)-dependent F-actin disruption in intestinal brush borders. *J. Cell. Biol.* 146 819–830. 10.1083/jcb.146.4.819 10459016 PMC2156144

[B19] Fong K. S. de Couet H. G. (1999). Novel proteins interacting with the leucine-rich repeat domain of human flightless-I identified by the yeast two-hybrid system. *Genomics* 58 146–157. 10.1006/geno.1999.5817 10366446

[B20] Ghoshdastider U. Popp D. Burtnick L. D. Robinson R. C. (2013). The expanding superfamily of gelsolin homology domain proteins. *Cytoskeleton* 70 775–795. 10.1002/cm.21149 24155256

[B21] Goshima M. Kariya K.-I. Yamawaki-Kataoka Y. Okada T. Shibatohge M. Shima F. (1999). Characterization of a Novel Ras-Binding Protein Ce-FLI-1 Comprising Leucine-Rich Repeats and Gelsolin-like Domains. *Biochem. Biophys. Res. Commun.* 257 111–116. 10.1006/bbrc.1999.0420 10092519

[B22] Gould C. J. Maiti S. Michelot A. Graziano B. R. Blanchoin L. Goode B. L. (2011). The formin DAD domain plays dual roles in autoinhibition and actin nucleation. *Curr. Biol.* 21 384–390. 10.1016/j.cub.2011.01.047 21333540 PMC3058777

[B23] He J. P. Hou P. P. Chen Q. T. Wang W. J. Sun X. Y. Yang P. B. (2018). Flightless-I Blocks p62-Mediated Recognition of LC3 to Impede Selective Autophagy and Promote Breast Cancer Progression. *Cancer Res.* 78 4853–4864. 10.1158/0008-5472.can-17-3835 29898994

[B24] Higashi T. Ikeda T. Murakami T. Shirakawa R. Kawato M. Okawa K. (2010). Flightless-I (Fli-I) Regulates the Actin Assembly Activity of Diaphanous-related Formins (DRFs) Daam1 and mDia1 in Cooperation with Active Rho GTPase. *J. Biol. Chem.* 285 16231–16238. 10.1074/jbc.M109.079236 20223827 PMC2871490

[B25] Kinosian H. J. Newman J. Lincoln B. Selden L. A. Gershman L. C. Estes J. E. (1998). Ca2+ regulation of gelsolin activity: binding and severing of F-actin. *Biophys. J.* 75 3101–3109. 10.1016/S0006-3495(98)77751-77753 9826630 PMC1299981

[B26] Kis-Bicskei N. Becsi B. Erdodi F. Robinson R. C. Bugyi B. Huber T. (2018). Tropomyosins Regulate the Severing Activity of Gelsolin in Isoform-Dependent and Independent Manners. *Biophys. J.* 114 777–787. 10.1016/j.bpj.2017.11.3812 29490240 PMC5984974

[B27] Kopecki Z. Arkell R. M. Strudwick X. L. Hirose M. Ludwig R. J. Kern J. S. (2011). Overexpression of the Flii gene increases dermal-epidermal blistering in an autoimmune ColVII mouse model of epidermolysis bullosa acquisita. *J. Pathol.* 225 401–413. 10.1002/path.2973 21984127

[B28] Kothakota S. Azuma T. Reinhard C. Klippel A. Tang J. Chu K. (1997). Caspase-3-generated fragment of gelsolin: effector of morphological change in apoptosis. *Science* 278 294–298. 10.1126/science.278.5336.294 9323209

[B29] Li J. Yin H. L. Yuan J. (2008). Flightless-I regulates proinflammatory caspases by selectively modulating intracellular localization and caspase activity. *J. Cell Biol.* 181 321–333. 10.1083/jcb.200711082 18411310 PMC2315678

[B30] Liu W. Sato A. Khadka D. Bharti R. Diaz H. Runnels L. W. (2008). Mechanism of activation of the Formin protein Daam1. *Proc. Natl. Acad. Sci. U.S.A.* 105 210–215. 10.1073/pnas.0707277105 18162551 PMC2224188

[B31] Liu W. Xie Y. Ma J. Luo X. Nie P. Zuo Z. (2015). IBS: an illustrator for the presentation and visualization of biological sequences. *Bioinformatics* 31 3359–3361. 10.1093/bioinformatics/btv362 26069263 PMC4595897

[B32] Liu Y. T. Yin H. L. (1998). Identification of the binding partners for flightless I, A novel protein bridging the leucine-rich repeat and the gelsolin superfamilies. *J. Biol. Chem.* 273 7920–7927. 10.1074/jbc.273.14.7920 9525888

[B33] Lu J. Dentler W. L. Lundquist E. A. (2008). FLI-1 Flightless-1 and LET-60 Ras control germ line morphogenesis in C. elegans. *BMC Dev. Biol.* 8:54. 10.1186/1471-213X-8-54 18485202 PMC2396608

[B34] Manchester K. L. (1995). Value of A260/A280 ratios for measurement of purity of nucleic acids. *Biotechniques* 19 208–210. 8527139

[B35] Matusek T. Gombos R. Szecsenyi A. Sanchez-Soriano N. Czibula A. Pataki C. (2008). Formin proteins of the DAAM subfamily play a role during axon growth. *J. Neurosci.* 28 13310–13319. 10.1523/JNEUROSCI.2727-08.2008 19052223 PMC6671601

[B36] Mohammad I. Arora P. D. Naghibzadeh Y. Wang Y. Li J. Mascarenhas W. (2012). Flightless I is a focal adhesion-associated actin-capping protein that regulates cell migration. *FASEB J.* 26 3260–3272. 10.1096/fj.11-202051 22581781

[B37] Nag S. Larsson M. Robinson R. C. Burtnick L. D. (2013). Gelsolin: the tail of a molecular gymnast. *Cytoskeleton* 70 360–384. 10.1002/cm.21117 23749648

[B38] Nag S. Ma Q. Wang H. Chumnarnsilpa S. Lee W. L. Larsson M. (2009). Ca2+ binding by domain 2 plays a critical role in the activation and stabilization of gelsolin. *Proc. Natl. Acad. Sci. U.S.A.* 106 13713–13718. 10.1073/pnas.0812374106 19666512 PMC2720848

[B39] Perrimon N. Smouse D. Miklos G. L. (1989). Developmental genetics of loci at the base of the X chromosome of Drosophila melanogaster. *Genetics* 121 313–331. 2499511 10.1093/genetics/121.2.313 PMC1203620

[B40] Pinson K. I. Dunbar L. Samuelson L. Gumucio D. L. (1998). Targeted disruption of the mouse villin gene does not impair the morphogenesis of microvilli. *Dev. Dyn.* 211 109–121. 10.1002/(sici)1097-0177(199801)211:1<109::aid-aja10>3.0.co;2-7 9438428

[B41] Pollard T. D. (2007). Regulation of actin filament assembly by Arp2/3 complex and formins. *Annu. Rev. Biophys. Biomol. Struct.* 36 451–477. 10.1146/annurev.biophys.35.040405.101936 17477841

[B42] Pope B. Way M. Weeds A. G. (1991). Two of the three actin-binding domains of gelsolin bind to the same subdomain of actin. Implications of capping and severing mechanisms. *FEBS Lett.* 280 70–74. 10.1016/0014-5793(91)80206-i 1849098

[B43] Schutt C. E. Myslik J. C. Rozycki M. D. Goonesekere N. C. Lindberg U. (1993). The structure of crystalline profilin-beta-actin. *Nature* 365 810–816. 10.1038/365810a0 8413665

[B44] Selden L. A. Kinosian H. J. Newman J. Lincoln B. Hurwitz C. Gershman L. C. (1998). Severing of F-actin by the amino-terminal half of gelsolin suggests internal cooperativity in gelsolin. *Biophys. J.* 75 3092–3100. 10.1016/S0006-3495(98)77750-77751 9826629 PMC1299980

[B45] Silacci P. Mazzolai L. Gauci C. Stergiopulos N. Yin H. L. Hayoz D. (2004). Gelsolin superfamily proteins: key regulators of cellular functions. *Cell Mol. Life Sci.* 61 2614–2623. 10.1007/s00018-004-4225-4226 15526166 PMC11924436

[B46] Spudich J. A. Watt S. (1971). The regulation of rabbit skeletal muscle contraction. I. Biochemical studies of the interaction of the tropomyosin-troponin complex with actin and the proteolytic fragments of myosin. *J. Biol. Chem.* 246 4866–4871. 4254541

[B47] Szatmari D. Bugyi B. Ujfalusi Z. Grama L. Dudas R. Nyitrai M. (2017). Cardiac leiomodin2 binds to the sides of actin filaments and regulates the ATPase activity of myosin. *PLoS One* 12:e0186288. 0.1371/journal.pone.0186288 10.1371/journal.pone.0186288 PMC5638494 29023566

[B48] Szikora S. Foldi I. Toth K. Migh E. Vig A. Bugyi B. (2017). The formin DAAM is required for coordination of the actin and microtubule cytoskeleton in axonal growth cones. *J. Cell Sci.* 130 2506–2519. 10.1242/jcs.203455 28606990

[B49] Toth M. A. Majoros A. K. Vig A. T. Migh E. Nyitrai M. Mihaly J. (2016). Biochemical Activities of the Wiskott-Aldrich Syndrome Homology Region 2 Domains of Sarcomere Length Short (SALS) Protein^∗^. *J. Biol. Chem.* 291 667–680. 10.1074/jbc.m115.683904 26578512 PMC4705388

[B50] Vig A. T. Foldi I. Szikora S. Migh E. Gombos R. Toth M. A. (2017). The activities of the C-terminal regions of the formin protein disheveled-associated activator of morphogenesis (DAAM) in actin dynamics. *J. Biol. Chem.* 292 13566–13583. 10.1074/jbc.M117.799247 28642367 PMC5566517

[B51] Witke W. Li W. Kwiatkowski D. J. Southwick F. S. (2001). Comparisons of CapG and gelsolin-null macrophages: demonstration of a unique role for CapG in receptor-mediated ruffling, phagocytosis, and vesicle rocketing. *J. Cell Biol.* 154 775–784. 10.1083/jcb.200101113 11514591 PMC2196452

[B52] Witke W. Sharpe A. H. Hartwig J. H. Azuma T. Stossel T. P. Kwiatkowski D. J. (1995). Hemostatic, inflammatory, and fibroblast responses are blunted in mice lacking gelsolin. *Cell* 81 41–51. 10.1016/0092-8674(95)90369-0 7720072

[B53] Yin H. L. Stossel T. P. (1979). Control of cytoplasmic actin gel-sol transformation by gelsolin, a calcium-dependent regulatory protein. *Nature* 281 583–586. 10.1038/281583a0 492320

[B54] Zhao X. Li G. Liang S. (2013). Several affinity tags commonly used in chromatographic purification. *J. Anal. Methods Chem.* 2013:581093. 10.1155/2013/581093 24490106 PMC3893739

